# The C-terminal domain of EFA6A interacts directly with F-actin and assembles F-actin bundles

**DOI:** 10.1038/s41598-019-55630-9

**Published:** 2019-12-16

**Authors:** Eric Macia, Mariagrazia Partisani, Hong Wang, Sandra Lacas-Gervais, Christophe Le Clainche, Frederic Luton, Michel Franco

**Affiliations:** 1 Université Côte d’Azur, Centre National de la Recherche Scientifique (CNRS), Institut de Pharmacologie Moléculaire et Cellulaire (IPMC), UMR 7275, 660 route des lucioles, 06560 Valbonne, France; 2 Institute for Integrative Biology of the Cell (I2BC), CEA, CNRS, Univ. Paris-Sud, Université Paris-Saclay, 91198 Gif-sur-Yvette, cedex France; 3 Université Côte d’Azur, Centre Commun de Microscopie Appliquée (CCMA), Parc Valrose, 06103 Nice, cedex 2 France

**Keywords:** Proteins, Cytoskeleton

## Abstract

The Arf6-specific exchange factor EFA6 is involved in the endocytic/recycling pathway for different cargos. In addition EFA6 acts as a powerful actin cytoskeleton organizer, a function required for its role in the establishment of the epithelial cell polarity and in neuronal morphogenesis. We previously showed that the C-terminus of EFA6 (EFA6-Ct) is the main domain which contributes to actin reorganization. Here, by *in vitro* and *in vivo* experiments, we sought to decipher, at the molecular level, how EFA6 controls the dynamic and structuring of actin filaments. We showed that EFA6-Ct interferes with actin polymerization by interacting with and capping actin filament barbed ends. Further, in the presence of actin mono-filaments, the addition of EFA6-Ct triggered the formation of actin bundles. In cells, when the EFA6-Ct was directed to the plasma membrane, as is the case for the full-length protein, its expression induced the formation of membrane protrusions enriched in actin cables. Collectively our data explain, at least in part, how EFA6 plays an essential role in actin organization by interacting with and bundling F-actin.

## Introduction

The ADP-ribosylation factor (Arf) family is comprised of 6 isoforms in mammalian cells, and is further subdivided into three classes based on primary sequence homology: Class I (Arf1/3), II (Arf4/5) and III (Arf6). Arf proteins are involved in intracellular transport, both in the endocytic-recycling and the biosynthesis-secretion pathways^1^. They are known to control lipid modifying enzymes and to recruit protein coats to facilitate the formation of membrane transport vesicles. Arf6, the most divergent isoform, has been shown to be involved in actin cytoskeleton organization at the cell surface in addition to its role in endocytic/recycling pathway. It has been implicated in the F-actin dependent formation of pseudopodia, lamellipodia, invadopodia as well as neurite and axon outgrowth^2–5^. At the molecular level, however, the role of Arf6 on actin cytoskeleton organization is not clearly understood. It seems that Arf6 effectors, such as Por1 and Nm-23H1, regulate the GDP/GTP cycle of the small G-protein Rac1^5,6^. The Arf6-GAP ASAP1 is also known to act as a F-actin organizer and, in particular, it has been implicated in invadopodia and podosome assembly^7^.

Activation of Arf proteins is carried out by the Sec 7 domain-containing protein family that catalyzes the necessary GDP/GTP exchange by facilitating the release of GDP, the rate limiting step of the activation. As upstream partners, these Arf Guanine Nucleotide Exchange Factors (ArfGEFs) define the timing and the site of Arf activation. They are divided in two classes: the large and the small ArfGEF^8^. The large GEF family consists of the GBF1/Gea and the Big/Sec 7 sub-families. They show activity on Arf class I and II but do not have activity on class III. Little is known about their mechanism of activation and subcellular localization. The small GEF family, only present in metazoans, is comprised of Arno/Cytohesin, Brag/GEP100 and EFA6 subfamilies. Their localization and their substrate specificity are relatively well described^8,9^. The cytohesin group localizes to the plasma membrane and the Golgi apparatus and preferentially activates Arf1^10,11^. Interestingly, the Pleckstrin Homology (PH) domain, present in the C-terminal region of the cytohesin protein, seems to act as an Arf6 effector leading to a molecular cascade in which the activation of Arf6 triggers that of Arf1^12–14^. Regarding the Brag/GEP100 proteins, their substrate specificity has not yet been clearly established *in vitro*. However, a signaling pathway at the plasma membrane, involving EGFR/Brag/Arf6 and AMAP1 has been shown to be implicated in breast cancer invasion and metastasis suggesting that Arf6 could be the physiological substrate^15^.

The human EFA6 family contains four tissue-specific EFA6 isoforms (EFA6A to D), which are encoded by four different genes. They share a common domain organization structure consisting of a highly divergent N-terminal domain with unknown functions, a central catalytic Sec 7 domain, a PH domain responsible for the plasma membrane localization by interacting with PIP_2_ and a C-terminus (Ct) containing a coiled-coil domain surrounded by two proline rich regions and involved in actin remodeling. Despite their homology and overlapping tissue distribution, it remains to determine whether the different EFA6 proteins play specific roles. EFA6 proteins appear to be the most Arf6-specific GEFs both *in vivo* and *in vitro*. They control the endocytic trafficking of different cargos such as GPCRs, the transferrin receptor, ion channels and the transport of membrane vesicles to form the apical lumen in epithelial cells^16–19^. EFA6 proteins are also able to reorganize actin cytoskeleton. Indeed, the exogenous expression of EFA6 leads to the formation of plasma membrane ruffles, but the mechanism that drives the formation of these F-actin rich structures is not clearly established. It could involve a downstream activation of Arf6 and Rac1^20^. However, EFA6 has also been shown to reorganize actin cytoskeleton independently of its nucleotide exchange catalytic activity. Indeed, the expression of a catalytically dead EFA6 protein that has been inactivated by point mutation or by Sec 7 domain deletion, a mutant mimicking the short isoform of EFA6A found in murine neural tissue^21^, leads to the lengthening of actin-rich dorsal microvillar protrusions^20,22^. Although we have demonstrated, by deletion experiments, that the Ct domain was responsible for these F-actin rich protrusions, the molecular mechanism that accounts for their formation is not understood.

Here we describe a novel property of the EFA6A C-terminal domain (EFA6-Ct) that explains its Arf6-independent regulation of F-actin dynamic and structure. We demonstrated that *in vitro* this domain regulates actin polymerization in a dose- and time-dependent manner and bundles actin filaments. We showed that the targeting of EFA6-Ct to the plasma membrane is necessary and sufficient to extend the microvilli-like actin-enriched structures. Furthermore, we observed that the PH domain, which we had previously demonstrated to directly interact with PIP_2_ and actin filaments, cooperates with the Ct to generate the full size filopodia-like plasma membrane extensions. Finally, at the ultrastructural level, EFA6A-PH-Ct appears to connect actin filaments to the plasma membrane in these filopodia-like structures that it generates.

## Results

### EFA6-Ct binds to actin filaments and induces their bundling *in vitro*

**Figure 1:**
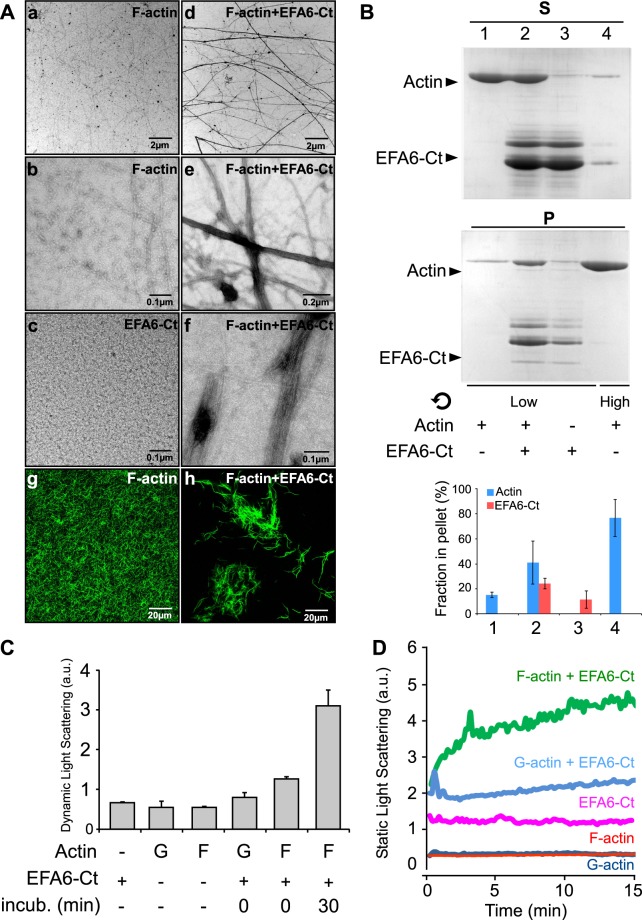
EFA6-Ct domain bundles F-actin *in vitro*. In all experiments, proteins were used at 4 µM. **(A)** Electron and fluorescent microscopy. F-actin alone (a,b,g) or EFA6-Ct alone (c) or the two proteins incubated together (d,e,f and h) were visualized by Transmission Electron Microscopy (a–f) or by fluorescent microscopy using FITC-phalloidin staining (g,h) **(B)** Co-sedimentation assay. As indicated, F-actin and EFA6-Ct were incubated separately or together for 15 min before low or high-speed centrifugation, then supernatants (S) and pellets (P) were analyzed by SDS-PAGE and Coomassie blue staining. The proportion of proteins recovered in the pellet was determined from four independent experiments. Means +/−SD are shown. Note that for each sample all the pellet and the half of the corresponding supernatant were loaded onto the SDS-gels. **(C)** Dynamic light scattering assay. As indicated, when present, G or F-actin was incubated with or without EFA6-Ct for 0 or 30 min. Quantification was determined from three independent experiments, means +/−SD are shown. **(D)** Static light scattering assay. G or F-actin were incubated alone (dark blue and red, respectively) or with EFA6-Ct (light blue and green). EFA6-Ct was incubated alone as a control (pink).

We had previously shown that the C-terminal domain of EFA6A (EFA6-Ct) was required *in vivo* to reorganize the actin cytoskeleton and to promote the lengthening of actin-rich plasma membrane extensions^20,22,23^. Here, we tested whether this Ct domain was able *per se* to control the structuring of actin filaments *in vitro*. The addition of purified N-terminally His-tagged EFA6-Ct to purified actin filaments induced the formation of actin bundles as observed by negative staining electron microscopy (Fig. 1A compare d to a). At higher magnifications we could observe the assembling of several mono-filaments induced by the addition of EFA6-Ct (Fig. 1A compare e and f to b). EFA6-Ct alone was shown as a control and no filament nor large aggregate were observed (Fig. 1A c). The formation of actin bundles was also visible by fluorescent microscopy after the addition of FITC-labeled phalloïdin (Fig. 1A compare h to g). The presence of EFA6-Ct in actin cables was assessed using low speed (16000x g) co-sedimentation (Fig. 1B). Indeed, the actin mono-filaments that are found in the pellet (~77%) after high speed centrifugation, only slightly sedimentate (~15%) at low speed (Fig. 1B, lanes 1vs4). However, when F-actin was incubated in the presence of EFA6-Ct, significant amounts of the two proteins (~41% and 24% respectively) were found in the low-speed pellet, indicating the presence of F-actin formed bundles that contained EFA6-Ct (Fig. 1B compare lane 2 to 1 and 3). By dynamic light scattering (DLS) we observed that the EFA6-Ct mediated formation of bundles occurred steadily over time (Fig. 1C). Indeed, the DLS signal was greatly increased after 30 min incubation of EFA6-Ct and F-actin together. It is noteworthy that neither polymerization of G-actin to form F-actin, nor the addition of EFA6-Ct to G-actin, affected the DLS signals (Fig. 1C). Finally, static light scattering was used to monitor the EFA6-Ct-induced formation of actin bundles in real time (Fig. 1D). When G-actin or F-actin was incubated without EFA6-Ct no increase in light scattering signal was observed (Fig. 1D red and dark blue traces, respectively) as expected in the absence of large structures. Similarly, when EFA6-Ct was incubated alone, no increase in the signal over time was observed (Fig. 1D pink trace). The addition of EFA6-Ct to G-actin led only to a slightly increased light scattering signal (Fig. 1D light blue trace). In contrast, in the presence of F-actin, EFA6-Ct caused a strong augmentation, as a function of time, in the size of the particles present in the sample thus confirming an EFA6-Ct induced formation of F-actin bundles (Fig. 1D green curve).

Altogether, these data demonstrate that the C-terminal domain of EFA6A is able to interact directly with and to bundle actin mono-filaments.

### *In vitro* EFA6-Ct inhibits the actin polymerization at the barbed ends

**Figure 2:**
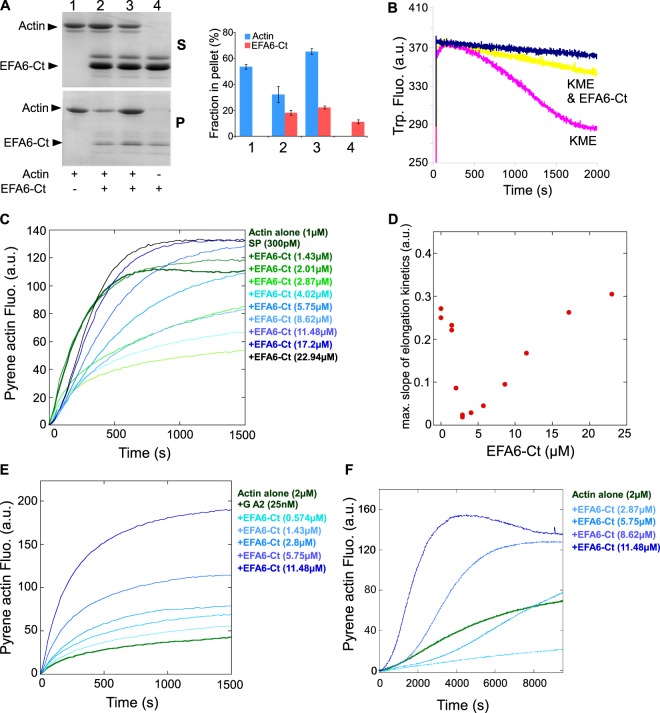
Regulation of actin polymerization by EFA6. **(A)** Co-sedimentation assay. When present, G-actin (4 µM) was polymerized with KME buffer for 45 min without (lane 1) or with EFA6-Ct (4 µm) added at the beginning (lane 2) or at the end (lane 3) of the 45 min incubation. As a control EFA6-Ct incubated alone in KME buffer (lane 4). After high speed centrifugation, supernatants (S) and pellets (P) were analysed by SDS-PAGE and Coomassie blue staining. The proportion of proteins recovered in the pellet was determined from five independent experiments, means +/−SD are shown. Note that for each sample all the pellet and the half of the corresponding supernatant were loaded onto the SDS-gels. **(B)** Tryptophan fluorescence measurement. G-actin (4 µM) was incubated alone (blue) or with KME buffer without (pink) or with EFA6-Ct (4 µM, yellow). **(C)** Actin polymerization was measured in the presence of 300 pM spectrin-actin seeds (SP), 1 µM MgATP-G-actin (10% pyrenyl-labeled) in the absence of presence of increasing concentrations of EFA6-Ct as indicated. **(D)** The maximum slopes of the kinetics shown in (**C**) are used as a readout for actin polymerization and plotted versus the concentration of EFA6-Ct. **(E)** Actin polymerization was measured in the presence of 25 nM gelsolin-actin complex (GA2), 2 µM MgATP-G-actin (10% pyrenyl-labeled) in the absence or presence of increasing concentrations of EFA6-Ct as indicated. **(F)** Spontaneous actin assembly, reflecting actin nucleation, was measured in the presence of 2 µM MgATP-G-actin (10% pyrenyl-labeled) in the absence or presence of increasing concentrations of EFA6-Ct as indicated. (**C**–**F**) Two independent dose dependence experiments were performed and showed the same results.

When EFA6-Ct was incubated with G-actin at the oncet of the polymerization reaction only about half of the actin was found in the pellet after high speed centrifugation (Fig. 2A compare lane 2 to 1 i.e. ~31% vs 54%). This observation suggests that the Ct could inhibit actin polymerization. However, as observed in Fig. 1, when EFA6-Ct was added after polymerization had been completed, F-actin was found in the pellet in the form of actin bundles (Fig. 2A lane 3). This result indicates that the Ct could inhibit actin polymerization but did not induce its disassembly. This polymerization inhibitory effect was investigated by the intrinsic tryptophan fluorescence change (Fig. 2B). In very low ionic strength conditions, no significant intrinsic fluorescence change of actin was observed in the absence of polymerization (Fig. 2B blue trace). In contrast, when G actin was incubated in polymerization buffer (KME), the fluorescence signal strongly decreased to reach a plateau after 1500 s (Fig. 2B pink trace). The addition of EFA6-Ct to the polymerization buffer (KME + EFA6-Ct) significantly slowed down the decrease in the fluorescence signal, confirming that EFA6-Ct inhibited actin polymerization (Fig. 2B yellow trace).

**Figure 3:**
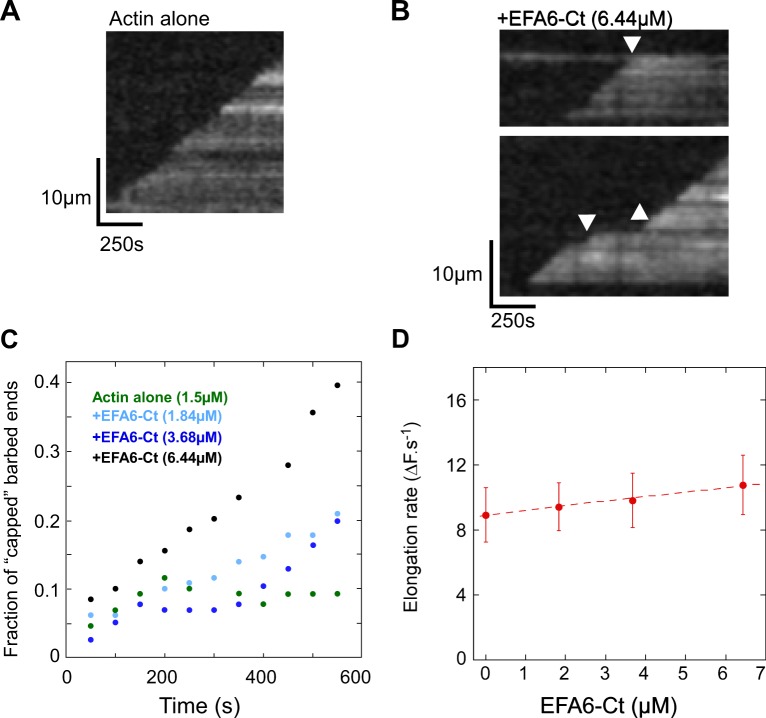
Direct real-time observation and quantification of the activity of EFA6-Ct on the elongation of single actin filaments by TIRF microscopy. (**A**,**B**) Kymographs representing the barbed end growth of single actin filaments in the absence (**A**) or presence of 6.44 µM EFA6-Ct (**B**). The fluorescence intensity was measured along the length of a single filament (vertical axis) for each frame of a time lapse (horizontal axis). In contrast with the linear elongation of the control filaments (**A**), pauses were observed in the presence of EFA6-Ct (arrows pointing down in B), indicating barbed-end capping events. Filaments occasionally restart after a pause (arrow pointing up in **B**) The kymograph shown in (**A**) corresponds to the filament 2 in the Supplemental Movie S1. The top and bottom kymographs shown in (**B**) correspond to the filaments 1 and 2 respectively in the Supplemental Movie S2. **(C)** Fraction of capped filaments over time in the presence of 0 µM EFA6-Ct (n = 43 filaments), 1.84 µM (n = 129), 3.68 µM (n = 116), 6.44 µM (n = 129). The fraction of capped barbed ends was obtained by calculating the following ratio from TIRF microscopy time lapses: (number of filaments showing a pause at the indicated time and concentration of EFA6-Ct)/(total number of analyzed filaments). **(D)** Elongation rate (subunits.s^−1^) of the filaments, during the growing periods, in the presence of the indicated concentrations of EFA6-Ct. Data are mean +/−SD.

To better characterize the mechanism by which EFA6-Ct inhibits actin polymerization, we combined kinetic assays in fluorescence spectroscopy and single filament observations in TIRF (Total Internal Reflection Fluorescence) microscopy. We first determined the ability of EFA6-Ct to specifically regulate the elongation of filament barbed ends (Fig. 2C). Increasing concentrations of EFA6-Ct affected the elongation of actin filament barbed ends following a biphasic response (Fig. 2D). At low concentration, EFA6-Ct strongly inhibited barbed end elongation, whereas higher concentrations gradually increased actin polymerization. In contrast, EFA6-Ct did not inhibit the elongation of the pointed ends, demonstrating that EFA6-Ct acts specifically at the barbed end (Fig. 2E). However, EFA6-Ct also stimulates actin assembly in these conditions. To unambiguously determine whether high concentrations of EFA6-Ct enhance the elongation of actin filament pointed ends or stimulate actin nucleation, we tested the effect of EFA6-Ct on spontaneous actin polymerization, which is sensitive to actin nucleation activities. This experiment clearly shows that EFA6-Ct nucleates actin assembly at high concentration (Fig. 2F). These observations indicate that, in addition to its ability to bind to the side of actin filaments, EFA6-Ct is also capable of two distinct activities: a main activity, which is to inhibit the barbed end elongation; and a second weaker activity, that is only seen at high concentration, which is to nucleate actin filaments. To validate this interpretation, we directly measured the elongation of single actin filaments by TIRF microscopy (Fig. 3). At low actin concentrations, actin filaments elongated only from their barbed ends. In these experiments, we observed that EFA6-Ct induced the appearance of occasional pauses in the linear elongation of actin filament barbed ends in a dose- and time-dependent manner (compare Fig. 3A,B, Movies S1 and S2). Indeed, the number of capped barbed ends increased with time and EFA6-Ct concentration (Fig. 3C), whereas the elongation rate between pauses was not affected (Fig. 3D). These observations support the view that EFA6-Ct inhibits barbed end elongation at low concentrations.

Altogether, our results demonstrate that EFA6 interacts with the side of actin filaments to induce their bundling. EFA6 also interacts with actin filament barbed ends to cap or orientate actin filaments respective to the membrane.

### Localization of EFA6-Ct to the plasma membrane is sufficient to induce actin-rich membrane extensions

**Figure 4:**
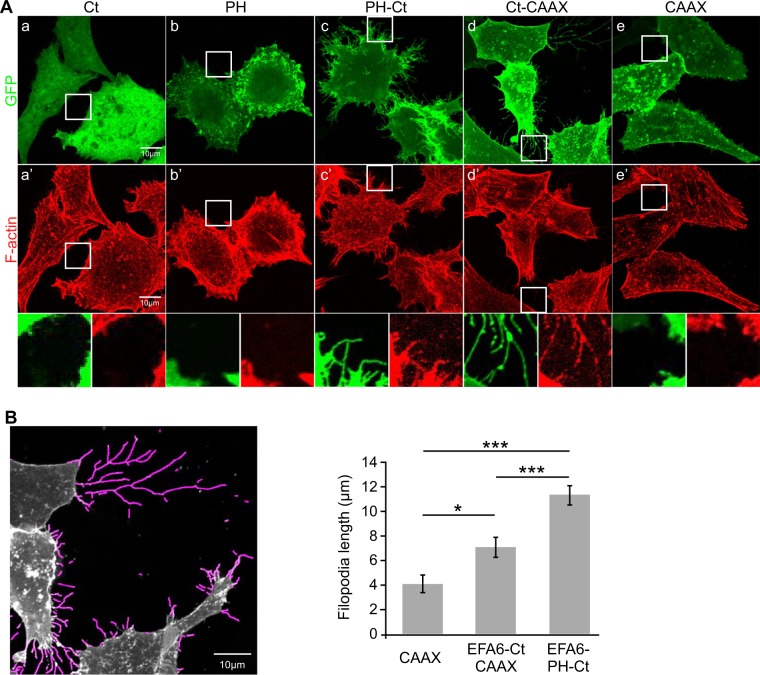
Effects of EFA6-Ct on actin cytoskeleton rearrangements in cells. (**A**) BHK-21 cells were transfected with pEGFP-EFA6ACt (a,a′); pEGFP-EFA6PH (b,b′); pEGFP-EFA6PH-Ct (c,c′); pEGFP-EFA6ACt-CAAX (d,d′); and pEGFP-CAAX (e,e′). After 24 h cells were fixed and labeled with Texas Red-conjugated phalloïdin to visualize F-actin reorganization. (**B)** Images obtained in (**A**) were treated with FiloQuant macro tools^41^. An example of processed EGFP-EFA6ACtCAAX expressing cells is given (left panel). The average length of the longest 10% filopodia is shown (right panel; n = 98,83 and 124 respectively). A Tukey test gives a p-value < 0.0001 between PH-Ct and other constructs and 0.02 between CAAX and EFA6ACtCAAX.

We then asked whether, when expressed in the cells, the isolated EFA6-Ct was able to induce the formation of plasma membrane extensions enriched in F-actin bundles. The expression of GFP-fused EFA6-Ct did not affect the morphology of BHK cells as observed by immuno-fluorescence (Fig. 4Aa,a′). The protein remained in the cytosol and the organization of the actin cytoskeleton was not modified. As previously observed^23^, the isolated PH domain localized to the plasma membrane without inducing filopodia like extensions (Fig. 4Ab,b′). However and as described previously^22^, the addition of this plasma membrane targeting PH-domain to the Ct led to a dramatic morphological change at the cell surface. Long microvillar F-actin based protrusions were formed (Fig. 4Ac,c′ and corresponding inset). To analyze the contribution of the PH domain to this phenotype, the Ct was targeted to the plasma membrane through a farnesylation modification driven by a CAAX box. Althought to a lesser extent than what we observed with the PH-Ct, the fanesylated-Ct promoted the lengthening of microvilli-like structures (Fig. 4Ad,d′,B for quantification). The CAAX fused to GFP was expressed as a control and no membrane filopodia like structure was observed (Fig. 4Ae,e′). Our data demonstrated that once targeted to the cell surface, the Ct is sufficient to trigger the formation of actin-rich plasma membrane extensions.

### EFA6-Ct induces and connects F-actin bundles to the plasma membrane

**Figure 5:**
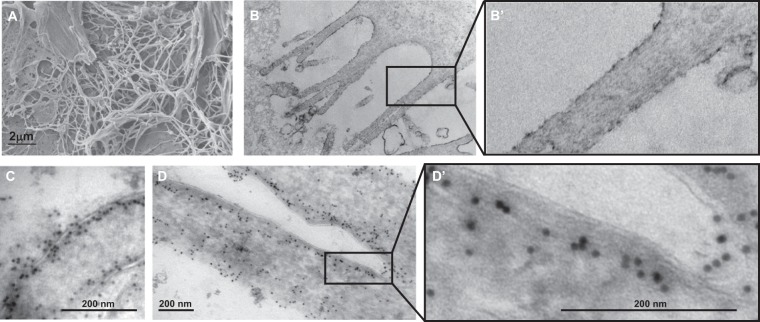
Analysis at the ultra-structural level of the EFA6APH-Ct-mediated cell membrane extensions. GFP-EFA6APH-Ct expressing BHK-21 cells were observed by Scanning Electron Microscopy **(A)** or by Transmission Electron Microscopy (**B** and **B**′ for inset). Localization of EFA6APH-Ct by immuno-electron microscopy (**C**,**D** and **D**′ for inset). BHK-21 cells expressing GFP-EFA6APH-Ct were fixed and processed for immuno-gold labeling. Sections were labeled with an anti-GFP antibody followed by protein A-15 nm gold.

Our next step was to use electron microscopy to look for EFA6 and F-actin in the microvilli-like membrane protrusions that had been induced by the expression of GFP-EFA6APH-Ct in BHK cells. As described previously^22^, the surface of EFA6-PH-Ct expressing cells was covered with long and thin membrane extensions, as observed by scanning electron microscopy (Fig. 5A). Using Transmission Electron Microscopy we observed that these structures are enriched in parallel F-actin cables (Fig. 5B,B′ at higher magnification). To determine the cellular localization of GFP-EFA6PH-Ct at the ultrastructural level, we performed immuno-gold labeling using an anti-GFP antibody. Electron micrographs revealed that the protein was located at the plasma membrane, distributed along and at the periphery of the protrusions and also along an electron dense structure that corresponds to the cortical actin bundles (Fig. 5C,D,D′ for higher magnification). Altogether, these data suggest that EFA6A-PH-Ct induced the formation of filopodia-like membrane extensions by assembling actin filaments in cables and connecting them to the plasma membrane.

## Discussion

**Figure 6:**
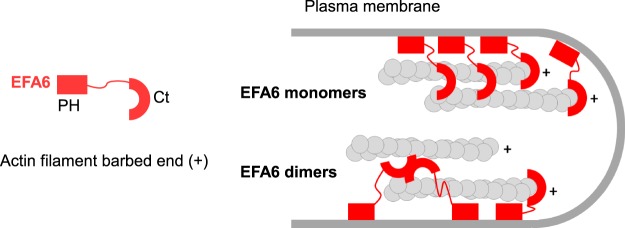
Schematic model of the molecular mode of action of EFA6-PHCt in the formation and the organization of F-actin bundles within plasma membrane protrusions.

Here, we have identified and characterized the intrinsic capability of the C-terminus of the Arf6 exchange factor EFA6 to interact with and regulate actin polymerization and assembly. We observed that EFA6-Ct is able to inhibit actin growth at the barbed ends by binding and capping F-actin. This effect is specific as EFA6-Ct does not inhibit actin elongation at the pointed ends. Moreover, the induction of pauses during the elongation of single actin filaments observed by TIRF video-microscopy confirmed that EFA6-Ct interacts with the barbed ends. At high concentrations, EFA6-Ct nucleates actin filaments, like other barbed end capping proteins^24^. These activities confirm the direct interaction between EFA6-Ct and actin filaments and suggest the existence of several actin-binding domains with different affinities and different functions. The relative importance of these domains is thus highly dependent on the local concentration of EFA6. EFA6-Ct can also bundle actin filaments, through actin filament side binding domain. We hypothesize that to bundle actin filaments, EFA6-Ct should carry two actin binding site or be able to form dimers *via* the presence of the coiled-coil domain in its centre (Fig. 6 for a model). Interestingly, the dual activities of capping and side-binding bundling are also found in EPS8^25,26^. It is noteworthy that cellular expression of EPS8^27^, similarly to EFA6 PH-Ct, promotes the assembly of actin-rich filopodia-like structures. We hypothesize that the interaction between EFA6-Ct and the barbed ends could be used to orientate the actin filaments respective to the plasma membrane. Indeed, the location of EFA6 will create a support point to give birth to a protrusion (Fig. 6) with the PH domain that localizes to specific PIP_2_-rich regions of the plasma membrane and the Ct that interacts with the barbed ends of the actin filaments. It is also important to note that the capping activity of EFA6 that we measured corresponds to very low-affinity binding of EFA6 to the barbed ends of actin filaments, as compared to true-capping-proteins^28^, suggesting that only a small fraction of barbed ends are capped for short periods of time in cells, leaving the majority of actin filaments free to polymerize. Therefore, our data favor a model in which membrane-bound EFA6 controls both the structure and the polarity of a polymerizing actin bundle against the plasma membrane to induce its protrusion.

EFA6A is highly expressed in the brain and enriched in the hippocampus^29,30^. In neuronal cells, it has been shown to be involved in the formation and the maturation of dendritic spines^3^. EFA6A depletion decreased spine formation, whereas its overexpression favors formation. EFA6 is suspected to regulate the formation of dendritic spines and their stability. The formation of spines and neurites requires tight coordination between the actin cytoskeleton and membrane delivery. Interestingly, neurite outgrowth was shown to be dependent on both the presence of the Sec 7 and the Ct domains^31^. We propose that EFA6 controls neurite outgrowth by regulating both the membrane delivery *via* the Arf6-mediated pathway^4,32^ and the stress forces *via* the Ct-induced structuring of actin filaments that we have identified in this study. Interestingly, Sironi *et al*. have reported (from a mouse embryo cDNA library) the existence of a short isoform of EFA6A lacking the Sec 7 domain and thus resembling to the so-called PH-Ct construct^21^. The authors demonstrated that the expression of this short isoform in PC12 cells induces the branching of dendrites under NGF-stimulation. This observation was in accordance with a previous study by Sakagami and colleagues who demonstrated that a GEF-defective mutant of EFA6A increased the formation of dendrites in primary hippocampal neurons^30^. In this study we have observed that the simple act of recruiting the Ct to the plasma membrane is sufficient to produce actin-rich plasma membrane extensions that resemble neurites. We propose that this Ct-mediated remodeling of actin is sufficient to generate constraint forces to initiate the formation of neurites. We have noticed that the cell membrane extensions obtained upon Ct expression are significantly longer when the Ct is translocated to the plasma membrane in the presence of the PH domain than by the farnesylation of the CAAX box. We do not think that the difference in the size is caused by a difference in the affinity of the two constructs for the plasma membrane lipids. Indeed, the two peptides were found to be located at the plasma membrane by immunofluorescence. Moreover, biochemical experiments have measured similar apparent affinities (in the micromolar range) between the EFA6A-PH domain and PIP_2_-containing membrane lipids, compared to the affinities between a farnesylated peptide and phospholipids^23,33^. We believe that the PH domain is preferentially localized to specialized domains of the plasma membrane. We previously demonstrated that the PH interacts weakly with F-actin and is localized to PIP_2_ and F-actin-containing structures of the plasma membrane, which could constitute the initial structures of the neuritis^22,23^. Thus, considered together, our work has highlighted a mechanism by which EFA6A and its short isoform act to regulate neurite spine formation.

EFA6 has been shown to be involved in other cellular functions where its ability to regulate F-actin dynamics appears to be crucial. EFA6 plays a key role in the establishment of epithelial cell polarity by facilitating the formation of tight junctions^34–36^. In this case, the EFA6-mediated coordination of Arf6 activation with F-actin reorganization is required. Indeed, neither the constitutively activated Arf6 by itself, nor the EFA6 PH-Ct domain alone, could assume the full function of EFA6, but in contrast, the co-expression of both is necessary and sufficient to phenocopy the effect of EFA6 on the formation and the function of the tight junctions^36^. EFA6 was precisely shown to stabilize the acto-myosin ring in which the tight junctions are anchored. What is the molecular pathway that starts with EFA6 and leads to actin reorganization? Arf6 has been molecularly connected to the actin cytoskeleton pathway *via* Rac1, Por1 and Asap1 but these effectors are not sufficient for the establishment of tight junctions since active Arf6 fails to establish the polarity. Among the EFA6A C-terminal interactors that have been identified thus far, only F-actin (this study) and α-actinin^16,30^ could be responsible for this remodeling of the actin cytoskeleton. Therefore, we propose that the formation of actin bundles due to the interaction between F-actin and EFA6A-Ct could participate in this stabilization.

In summary, the purpose of this study was to find out how the C-terminal domain of EFA6A was able to reshape actin filaments. We identified and characterized a direct interaction between this domain and actin filament barbed ends. This domain is also involved in the formation of actin bundles. In parallel, we observed that the C-terminal was capable of modulating the rate of actin polymerization, particularly at the barbed ends, suggesting a more complex and complete role on F-actin dynamics. We propose that these actin structuring activities of the Ct domain, reinforced by its direct interaction with α-actinin, play a key role in numerous EFA6A-mediated cellular functions and particularly in the formation and the branching of neurites.

### Experimental procedures

#### DNA constructs

Plasmids encoding EGFP-EFA6PH-Ct, EGFP-EFA6Ct, GST-EFA6Ct have been described elsewhere^17,23^. EGFP-CAAX was a gift from Dr. A. Benmerah. The nucleotide sequence encoding the last C terminal 15 residues of KRas4B, which contain the CAAX box, was fused to the sequence encoding EFA6ACt by PCR and cloned into pEGFPC3 (Clontech) to obtain EGFP-EFA6Ct-CAAX. Sequence encoding residues 490–645 (Ct)^20^ was obtained by PCR and cloned into the pET8c plasmid (Novagen) for in-frame fusion with a Hexa-His-tag at the N terminus.

#### Cell culture, reagents and antibodies

Baby hamster kidney cells (BHK) were grown in BHK-21 medium (Gibco-BRL), containing 5% FCS, 10% tryptose phosphate broth, 100 U/mL penicillin, 100 μg/mL streptomycin and 2 mM L-glutamine as previously described^23^. The following antibodies were used: mouse monoclonal antibody (mAb) against the vsv-g epitope (clone P5D4, Roche Diagnostics GmbH, Mannheim, Germany), rabbit antiserum against GFP (Clontech), mouse monoclonal anti-actin (clone AC40, Sigma), mouse mAb against Arf6 (provided by S. Bourgoin, Sainte-Foy, Canada). FITC-conjugated phalloïdin was from Molecular Probes, FITC and Texas-Red-conjugated antibodies were from Jackson ImmunoResearch.

#### Expression and purification of recombinant proteins

His-EFA6Ct and GST-EFA6Ct were prepared according manufacturer’s instructions (Qiagen and GE healthcare, respectively).

#### Actin purification and polymerization

Monomeric G-actin was purified from rabbit muscle as described^37^, stored at 4 °C in G buffer (5 mM Tris/HCl pH 7.5; 0.2 mM ATP; 1 mM dithiothreitol; 0.1 mM CaCl_2_; 0.01% NaN_3_). Polymerization was triggered by addition of KME buffer (100 mM KCl,1 mM MgCl_2_, 0.2 mM EGTA) and was followed in real time by measurement of tryptophan fluorescence (ex 300 nm, em 335 nm) as previously described^38^.

#### Fluorescent pyrenyl-actin polymerization assay

Actin polymerization was monitored by the increase in fluorescence of 10% pyrenyl-labeled actin as previously described^24^. Actin polymerization was induced by the addition of 50 mM KCl, 1 mM MgCl_2_, and 0.2 mM EGTA to a solution of 10% pyrenyl-labeled CaATP-G-actin containing the proteins of interest. Fluorescence measurements were carried out in a Safas Xenius model FLX (Safas, Monaco) spectrophotometer. For kinetic experiments, 300 pM spectrin-actin seeds were added to the reaction for barbed end elongation measurements, and 25 nM gelsolin-actin (1:2) complexes were added for pointed end elongation measurements.

#### Actin sedimentation

For low speed sedimentation, actin was centrifuged at 16000 g for 5 min (Eppendorf 5415 R). High speed sedimentation was done at 150000 g for 15 min (Beckman TLA100.1 rotor).

#### Transmission electron microscopy (TEM)

Transmission Electron Microscopy imaging was performed essentially as previously described^23^. Briefly, samples containing purified F-actin (5 µM) were incubated for 5 min at room temperature with purified His-EFA6-Ct (2 µM) in G-KME buffer. Samples were deposited on glow discharge carbon coated grids and negatively stained with 1% aqueous uranyl acetate. They were observed with a JEOL 1400 transmission electron microscope at 100 kV. Acquisitions were made with a Morada Olympus CCD camera and magnifications were comprised between 10 and 200 thousand times (Nominal magnifications in Fig. 1A, a: 12.000x; b: 100.000x; c: 100.000x; d: 10.000x; e: 100000x and f: 200.000x).

For ultrastructural analysis, cells were fixed in 1.6% glutaraldehyde in 0.1 M phosphate buffer, rinsed in 0.1 M cacodylate buffer, post-fixed for 1 h in 1% osmium tetroxide and 1% potassium ferrocyanide in 0.1 M cacodylate buffer to enhance the staining of membranes. Cells were rinsed in distilled water, dehydrated in alcohols and lastly embedded in epoxy resin. Contrasted ultrathin sections (70 nm) were analyzed using the microscope mentioned above. For immunogold staining, cells were fixed with 4% paraformaldehyde, 0.1% glutaraldehyde in 0.1 M phosphate buffer (PB; pH 7.4) for 2 h and were processed for ultracryomicrotomy according to a slightly modified Tokuyasu method^39^. In brief, a cell suspension was spun down in 10% gelatin. After immersion in 2.3 M sucrose (in 0.1 M PB, pH 7.4) overnight at 4 °C, the samples were rapidly frozen in liquid nitrogen. Ultrathin (70 nm thick) cryosections were prepared with an ultracryomicrotome (Leica EMFCS, Austria) and mounted on formvar-coated nickel grids (Electron Microscopy Sciences, Fort Washington, PA, USA). Immunostaining samples were processed with an automated immunogold labeling system Leica EM IGL as following: the grids were incubated successively in PBS containing 50 mM NH_4_Cl (2x, 5 min), PBS containing 1% BSA (2x, 5 min.), PBS containing the relevant primary antibody in 1% BSA for 1 h, PBS containing 0.1% BSA (3x, 5 min), PBS containing 1% BSA and 15 nm colloidal gold conjugated protein AG (CMC, University Medical Center, Utrecht, The Netherlands), PBS containing 0.1% BSA for 5 min, PBS for 5 min twice. Lastly, the samples were fixed for 10 min with 1% glutaraldehyde, rinsed in distilled water and were contrasted with a mixture of methylcellulose/sucrose and 0.3% uranyl acetate on ice. After having been dried in air, sections were examined under the JEOL 1400 transmission electron microscope.

#### Scanning electron microscopy (SEM)

For SEM the cells were fixed in 1.6% glutaraldehyde in 0.1 M phosphate buffer, rinsed in 0.1 M cacodylate buffer, rinsed in distilled water and dehydrated with a series of ethanol solutions of increasing concentrations. Samples were then dried using hexamethyldisilane (Carl Roth, Karlsruhe, Germany) and sputter-coated with a 3 nm gold-palladium coating prior to analysis with the scanning electron microscope, JEOL 6700 F. The images were collected at low voltage, 1–3 kV.

#### Observation of actin cables formation by static light scattering, dynamic light scattering (DLS) and fluorescence microscopy

Actin bundle formation was monitored in real time by static light scattering using Jasco FP8300 (ex/em 550^40^, by DLS using a Dynapro MSX instrument (Protein Solutions) equipped with a Peltier temperature controller and visualized by microscopy using fluorescent FITC-phalloidin.

#### Observation and measurement of single actin filament elongation by TIRF microscopy

Our protocol is a modification of the protocol used to study vinculin activity^24^. To force the filaments to grow on the surface of the coverslip, we first irradiate coverslips with deep UV for 1 min and incubate them with 10% biotin-labeled PLL-PEG (ref x2) for 1 h at room temperature. The coverslips were then washed extensively with water and dried under a nitrogen stream. Flow cells containing 40–60 µL of liquid were prepared by sticking a PLL-PEG-coated coverslip to a slide with double face adhesive spacers. The chamber was first incubated with 0.1 mg/mL streptavidin for 5 min and washed with washing buffer (10 mM Tris pH 7.8, 200 µM ATP, 1 mM DTT, 1 mM MgCl_2_, 25 mM KCl, 0.1 mM CaCl_2_). The chamber was then saturated with 10% BSA for 5 min and washed with washing buffer. The final reaction (in 10 mM Tris-HC,l 25 mM KC,l 1 mM MgCl_2_, 0.2 mM EGTA, 0.1 mM CaCl2, 0.2 mM ATP, 0.4% Methycellulose, 5 mM DABCO, 20 mM DTT) was then injected into the chamber. A typical reaction was composed of 1.5 µM 5% Alexa488/5% Biotin-labeled-MgG-actin in 5 mM Tris pH 7.8, 200 µM ATP, 0.8% methyl-cellulose, 1 mM 1,4-diazabicyclo(2,2,2)-octane (DABCO), 25 mM KCl, 1 mM MgCl_2_, 200 µM EGTA, 10 mM DTT supplemented with EFA6-Ct or not. Finally, we sealed the flow chamber with VALAP (a mixture of vaseline, lanolin, and paraffin) and observed the reaction on an Olympus AX71 inverted microscope equipped with a 60 × (numerical aperture 1.45) objective (Olympus) and a Blues 473-nm laser (Cobolt). The time-lapse videos were acquired by Metamorph and subsequently analyzed by ImageJ.

## Supplementary information

Movie-S1

Movie-S2
